# Pathway-Based Integrative Analysis of Metabolome and Microbiome Data from Hepatocellular Carcinoma and Liver Cirrhosis Patients

**DOI:** 10.3390/cancers12092705

**Published:** 2020-09-21

**Authors:** Boram Kim, Eun Ju Cho, Jung-Hwan Yoon, Soon Sun Kim, Jae Youn Cheong, Sung Won Cho, Taesung Park

**Affiliations:** 1 Interdisciplinary Program in Bioinformatics, Seoul National University, Seoul 08826, Korea; mari911@snu.ac.kr; 2 Department of Internal Medicine and Liver Research Institute, Seoul National University College of Medicine, Seoul 03080, Korea; creatio3@snu.ac.kr (E.J.C.); yoonjh@snu.ac.kr (J.-H.Y.); 3 Department of Gastroenterology, Ajou University School of Medicine, Suwon 16499, Korea; soonsunkim@aumc.ac.kr (S.S.K.); jaeyoun620@aumc.ac.kr (J.Y.C.); sungwoncho@aumc.ac.kr (S.W.C.); 4 Department of Statistics, Seoul National University, Seoul 08826, Korea

**Keywords:** hepatocellular carcinoma, cirrhosis, microbiome, metabolome, multiomics, integration, metabolic pathway

## Abstract

**Simple Summary:**

Hepatocellular carcinoma (HCC) is one of the diseases associated with human microbiome. The human microbiome is known to affect human disease through the metabolites. The aim of this study was to identify the pathways associated with HCC by integrating microbiome and metabolomic data via a novel pathway-based integrative method named HisCoM-MnM representing Hierarchical structural Component Model for pathway analysis of Microbiome and Metabolome. Application of HisCoM-MnM to datasets from HCC and liver cirrhosis (LC) patients successfully identified HCC-related pathways related to cancer metabolic reprogramming along with the significant metabolome and metagenome that make up those pathways.

**Abstract:**

Aberrations of the human microbiome are associated with diverse liver diseases, including hepatocellular carcinoma (HCC). Even if we can associate specific microbes with particular diseases, it is difficult to know mechanistically how the microbe contributes to the pathophysiology. Here, we sought to reveal the functional potential of the HCC-associated microbiome with the human metabolome which is known to play a role in connecting host phenotype to microbiome function. To utilize both microbiome and metabolomic data sets, we propose an innovative, pathway-based analysis, Hierarchical structural Component Model for pathway analysis of Microbiome and Metabolome (HisCoM-MnM), for integrating microbiome and metabolomic data. In particular, we used pathway information to integrate these two omics data sets, thus providing insight into biological interactions between different biological layers, with regard to the host’s phenotype. The application of HisCoM-MnM to data sets from 103 and 97 patients with HCC and liver cirrhosis (LC), respectively, showed that this approach could identify HCC-related pathways related to cancer metabolic reprogramming, in addition to the significant metabolome and metagenome that make up those pathways.

## 1. Introduction

Recent advances in throughput and improvement in the accuracy of metagenomic sequencing have enabled active research regarding specific microbiomes associated with diseases. The human microbiome has been well implicated in the progression of various diseases. Such diseases include infectious diseases [1,2], metabolic disorders (e.g., obesity [3], type 2 diabetes [4]), respiratory diseases [5], autoimmune disorders [6], mental or psychological illnesses [7,8], gastrointestinal disease (e.g., inflammatory bowel disease [9]), and liver diseases, such as alcoholic liver disease, nonalcoholic fatty liver disease, cirrhosis, and hepatocellular carcinoma (HCC) [10,11,12]. Despite our extensive findings, little progress has been made in discovering the pathways that underlie these diseases, and the role in the pathophysiology of disease. Therefore, the use of other types of biological data can enhance the understanding of biological processes, and functions of the microbiome, in specific disease phenotypes. There are now many studies that associate microbiome data with genomic, epigenomic, transcriptomic, and metabolomic data in humans [13]. Among the numerous types of omics data, metabolome have additionally improved our ability to understand the structure and function of the microbiome, in numerous disease states [14,15]. Because the metagenome contains more metabolic genes than those found in the entire human genome, it plays an essential role in human physiology, with regard to factors such as enzymes, metabolites, and biological pathways. Several recent studies have now paired microbiome and metabolomic data to identify distinct mechanisms connecting the microbiome to human disease. For example, Noecker et al. [16] used a community-based metabolite potential score to estimate relative metabolic creation or depletion capacity of the microbiome, and also attempted to identify key microbial species. This group also compared predicted and measured metabolites, to identify interactions between humans and the microbiome.

However, many challenges remain. Most studies have analyzed microbiome and metabolomic data independently, or focused only on analyzing statistical associations between these two data types, underscoring a need for methods of integration. In this study, we present such a new integration method, Hierarchical structural Component Model for pathway analysis of Microbiome and Metabolome (HisCoM-MnM). This model extends our previously developed Pathway-based approach using HierArchical structure of collapsed RAre variant Of High-throughput sequencing data (PHARAOH) method [17]. PHARAOH is a pathway analysis method for rare genetic variants that analyzes pathways using a single hierarchical model consisting of collapsed gene-level summaries and pathways. This method was later extended to various data types such as common genetic variants, and miRNA and mRNA expression [18,19,20,21,22].

In the current work, we used the main framework of PHARAOH to construct a hierarchical component model for integrating microbiome and metabolomic data, using pathway information. Based on HisCoM-MnM, we aimed to identify disease-related pathways, and elucidate the underlying “crosstalk” between human microbiome and metabolome, by discovering pathways associated with pathogenic metagenome and metabolome specific to distinct diseases, using a single model to consider potential correlations, between pathways, and between microbiome and metabolomic data, using a ridge-type penalty. HisCoM-MnM allows us to understand the relationships between different biological “layers,” based on pathway information related to specific phenotypes.

Here, we applied our HisCoM-MnM model to analyze microbiome and metabolomic data from HCC and liver cirrhosis (LC) patients [23,24]. HCC is the most common type of primary liver cancer, making up > 90% of cases, and is the major leading cause of cancer-related death worldwide [25]. To identify biomarkers for the early detection of HCC, many studies have analyzed its possible associations with the human microbiome [11]. We further aimed to identify HCC-related pathways and KEGG orthologs and metabolites that could discriminate HCC from LC, using HisCoM-MnM, thus providing biological relevance between HCC and LC, in the context of pathways.

## 2. Results

### 2.1. Baseline Characteristics

The baseline characteristics of subjects in the HCC and LC groups are shown in Table 1. Subjects in both groups were similar in age and gender, although males were slightly predominant. Subjects in the LC group were more likely to have decompensated liver disease than the HCC group, wherein most of the subjects had compensated liver disease, and all HCC subjects had hepatitis B virus-related liver disease. Serum α-fetoprotein (AFP) levels were higher in the HCC than LC group. About 80% of the HCC cases were of stage I and II, according to the 7th American Joint Committee on Cancer staging system [26].

### 2.2. Pathway Analysis of HCC and LC Data

We next applied HisCoM-MnM to microbiome and metabolomic data from HCC and LC patients. Hospital information, age, gender, and AFP were included as covariates in the pathway analysis. A total of 111 pathways were commonly mapped between metagenomic and metabolomic data. For these 111 pathways, 2236 unique KEGG orthologs, and 74 unique metabolites, were mapped. HisCoM-MnM successfully found 39 significant pathways with *p*-values smaller than 0.01, after Bonferroni adjustment. Among these 39 pathways, most were related to metabolism (24 pathways), with other pathways related to organismal systems (5 pathways), human diseases (4 pathways), environmental information processing (3 pathways), cellular processes (2 pathways), and genetic information processing (1 pathway), according to classifications by the KEGG pathway database (Table 2). Among four human disease-related pathways, central carbon metabolism in cancer (KEGG pathway map05230) was in a cancer-related subcategory (Table 2).

**Figure 1:**
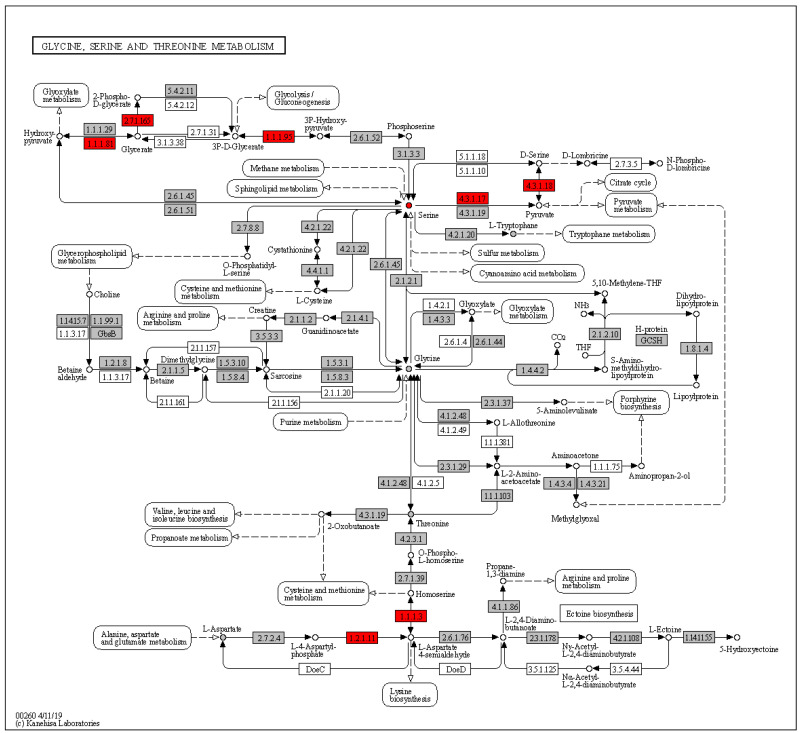
Glycine, serine, and threonine metabolic pathways. Circles represent compounds and rectangles are enzymes. The KEGG orthologs and metabolites that were significantly related with HCC are marked in red, otherwise in grey.

The central carbon metabolism cancer pathway contained a total of 25 KEGG orthologs, and metabolites, in HisCoM-MnM. Among these 25, only serine was significant, with a nominal *p*-value of 0.024. To delineate the details of serine metabolism in this pathway, we checked the results of pathways related to metabolic pathway reprogramming of cancer cells, out of our total 111 pathways, finding that the serine metabolism pathway related to another pathway named glycine, serine, and threonine metabolism. This pathway was significant (nominal *p*-value of 2.0 × 10^−4^), and the involvement of serine in this pathway was also significant, with a nominal *p*-value of 0.041. In Figure 1, we can see the entire metabolic pathways of glycine, serine, and threonine, as generated by KEGG Mapper. Among metabolites, only serine (red circle) showed significant results, and several KEGG orthologs marked with red rectangles showed significant results. Among significant KEGG orthologs, *PHGDH* (K00058), which encodes a metabolic enzyme involved in serine synthesis, was identified to be significant in HCC, with a nominal *p*-value of 0.029.

In addition, we identified other amino acid and lipid-metabolic pathways related to HCC, as well as the choline metabolism in cancer (map05231) pathway (Table 3). Choline metabolism in cancer is another significant (*p*-value of 0.0025) cancer-related pathway. We also found glycerophospholipid metabolism (map00564) as a significant result, with a *p*-value of 0.0012, consistent with previous findings [27].

### 2.3. Comparison of HisCoM-MnM to HisCoM-Single Omics

**Figure 2:**
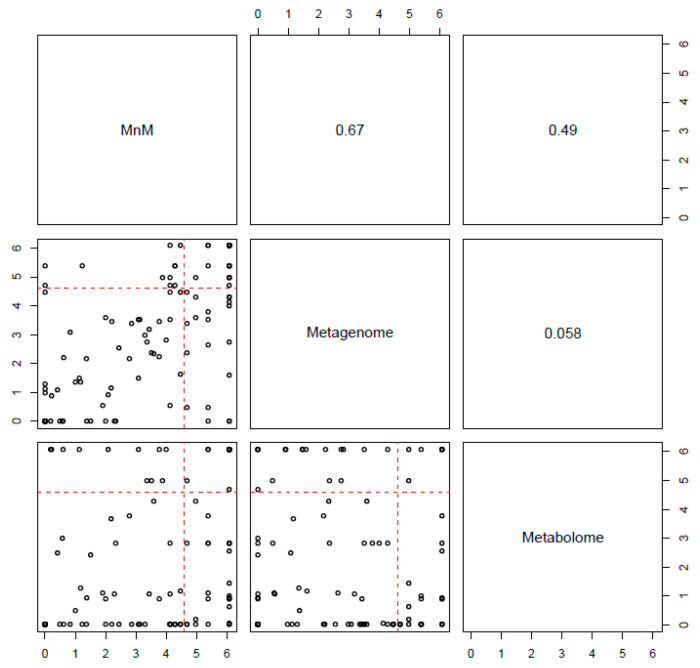
Pairwise scatter plot of 111 pathways’ *p*-values calculated by each method. The upper panel shows the Spearman’s rank correlation coefficient, and the lower panel is a scatter plot of the 111 pathways’ *p*-values. These *p*-values, from each method, were adjusted by Bonferroni correction, and a cutoff of 0.01 is represented by the red dotted lines.

**Figure 3:**
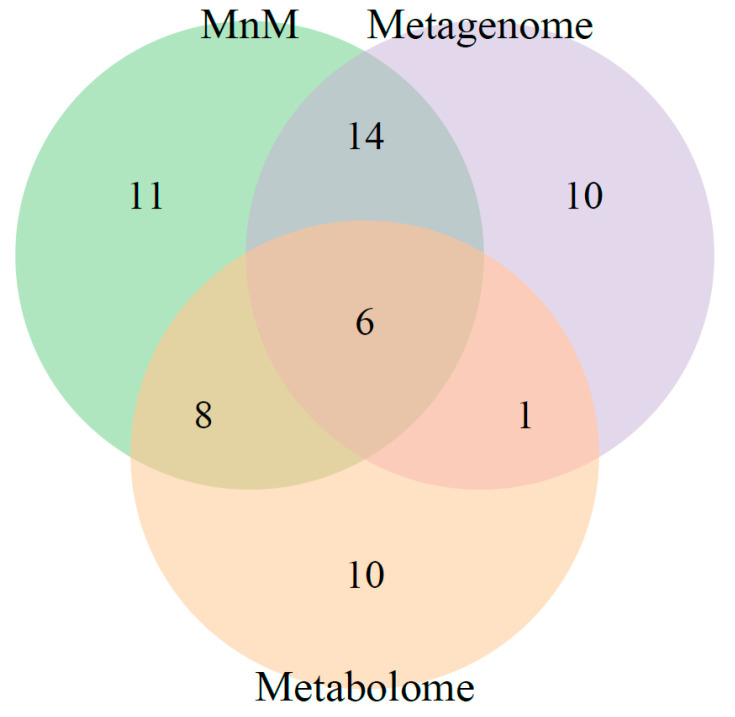
Venn diagram summarizing the number of significant pathways shared among three methods, HisCoM-MnM, metagenome, and metabolome. The number of significant pathways in the Venn diagram was determined by Bonferroni-corrected *p*-values with a cutoff 0.01.

Next, we confirmed whether the multiomics approach, HisCoM-MnM, could find significant results, as well as interpretive aspects, when compared to results from single omics data analysis. First, we applied HisCoM to the same HCC and LC metabolomic and metagenomic data used by HisCoM-MnM. Hospital information, age, gender, and AFP were also used as covariates, and the 111 pathways analyzed by HisCoM-MnM were also analyzed by single omics analysis. Figure 2 shows a pairwise scatter plot comparing *p*-values of the 111 pathways, as calculated by each method. The correlation between HisCoM-MnM and metagenomic data was higher than that between HisCoM-MnM and metabolomic data, showing that HisCoM-MnM and metagenomic data tend to have more similar pathway results than HisCoM-MnM and metabolomic data. Figure 3 is a Venn diagram showing that six significant pathways were shared between HisCoM-MnM, metagenome, and metabolome, including a cancer-related pathway, central carbon metabolism in cancer (as described earlier). In particular, HisCoM-MnM identified 11 significant pathways not found by the metagenomic and metabolomic single omics model. Many of these were previously observed in HCC, including fatty acid metabolism [28], valine, leucine, and isoleucine biosynthesis [28], selenocompound metabolism [29], and the forkhead box, class O (FOXO) signaling pathway [30]. These HCC-related pathways were only identified by HisCoM-MnM, but not by single omics analysis. Based on the results so far, HisCoM-MnM has successfully identified important pathways well known to associate with HCC and LC.

## 3. Discussion

This study was designed to identify HCC-related pathways, and KEGG orthologs and metabolites that discriminate HCC from LC. To utilize both microbiome and metabolomic data, we proposed a novel pathway analysis method, HisCoM-MnM, for integrating microbiome and metabolomic data sets. HisCoM-MnM has several advantages over traditional approaches, in that it simultaneously performs not only pathway analysis, but also analyzes components of pathways, such as metabolic genes and metabolites. This method also considers correlations between pathways and between its components. To consider correlations, HisCoM-MnM applies a ridge-type penalization approach. As a result, HisCoM-MnM identified pathways well linked to HCC, suggesting that the development of HCC is more related to amino acid and lipid metabolic reprogramming, compared to LC.

One of the significant pathways was central carbon metabolism in cancer [31]. It is well known that central metabolic pathways operating in cancer cells are fundamentally different from normal cells, in that cancer cells must generate the additional energy and biomass required to support rapid cell division. Therefore, cancer cells use different metabolic pathways to meet increased energy and biosynthesis demands during the process of tumor progression. One well-characterized metabolic phenotype observed in cancer cells is the Warburg effect, a shift from aerobic adenosine triphosphate (ATP) production, through mitochondrial oxidative phosphorylation (OXPHOS), to ATP production through anaerobic glycolysis, which converts glucose to lactate (without OXPHOS). Paradoxically, the Warburg effect is seen even under normal oxygen concentrations, and is widely observed in human cancers, including HCC [32].

We also identified the pathway named glycine, serine and threonine metabolism. It is well known that the serine/glycine biosynthetic pathway plays a crucial role in cancer cell proliferation. Serine and glycine are amino acids that are incorporated into proteins, and also serve as precursor molecules and substrates for cell growth and proliferation [33]. This shared biosynthetic pathway starts by oxidizing the glycolytic intermediate 3-phosphoglycerate into 3-phospho-hydroxy- pyruvate, catalyzed by the enzyme phosphoglycerate dehydrogenase (*PHGDH*, KEGG component K00058). 3-Phospho-hydroxypyruvate is subsequently metabolized into serine, via phosphoserine aminotransferase 1 (*PSAT1*, K00831), and phosphoserine phosphatase (*PSPH*, K01079) [34] and serine hydroxymethyltransferase (*SHMT*, K00600) next catalyze the conversion of serine into glycine. Moreover, HCC cells are known to support liver tumorigenesis by increasing serine and glycine synthesis by upregulating *PHGDH*, and other metabolic genes [35]. This is consistent with our findings that we found serine and *PHGDH* as significant results.

In addition, the dysregulation of amino acids (e.g., glutamine, glutamate, glycine, and aspartate), fatty acids, and bile acids, in HCC versus LC, was reported in previous studies [27,28,36,37,38,39]. In particular, we also found amino-acid- and lipid-metabolism-related pathways to be significant in our study. These findings reflect the Warburg effect, involving a metabolic shift for energy production.

Another significant cancer-related pathway was choline metabolism in cancer. Choline is an essential component of cell membranes, and abnormal choline metabolism is a metabolic feature of oncogenesis and tumor progression [40]. In particular, levels of glycerophosphocholine, phosphatidylcholine, and choline were reported to be elevated in HCC, compared to LC. In addition, increases in the phospholipid, phosphatidylcholine, were observed in early-stage HCC. These findings suggest dysregulation of glycerophospholipid metabolism in the early stages of HCC development. In this study, most HCC patients were of early-stage disease, with about 80% in stage I and II (Table 1). Our study also showed a significant result for glycerophospholipid metabolism, consistent with previous findings [27].

Compared to single omics approaches, HisCoM-MnM has the advantages of identifying significant pathways and aspects of interpretation. By a multiomics approach, HisCoM-MnM identified major HCC-related pathways not found by single omics approaches. For example, HisCoM-MnM detected significant pathways, such as metabolic reprogramming of fatty acids and amino acids. Furthermore, there were pathways known to relate to the suppression of cancer. One of the significant pathway was selenocompound metabolism. The potential for the anticancer effects of the micronutrient element selenium was demonstrated previously [29]. Another significant pathway, FOXO signaling, was reported to play a role in HCC growth inhibition by activating transcription of a thioredoxin-interacting protein considered to be a tumor suppressor [30].

In addition, HisCoM-MnM is a highly utilized method. First, HisCoM-MnM is flexible to the methods used to obtain the pathway-matching information. In this study, we used 16S rRNA-based microbiome data, and PICRUSt, to predict putative functions of the microbiome. Because PICRUSt provides KEGG-based information, we obtained metabolic gene information, as KEGG orthologs matched to KEGG pathways. To integrate metabolomic and microbiome data, through pathway information, we annotated metabolites to KEGG compound IDs, for pathway matching. Although we used the KEGG database for pathway matching, other pathway database and other types of data, such as shotgun sequencing data or untargeted metabolomic data, can also be used, because HisCoM-MnM only requires matching information between pathways, and components of pathways. Second, while HisCoM-MnM was developed for analyzing hepatocellular carcinoma and liver cirrhosis data, it can be applicable to any complex diseases associated with the microbiome.

There are some limitations to the present study. First, this study mainly focused on hepatitis B virus (HBV)-related HCC patients, with no patients with etiologies such as HCV and non-viral. Therefore, further research is required to confirm the generalizability of our results to other HCC etiologies. Second, the information in the microbiome and metabolomic data has not been fully utilized, due to limitations of the annotation. As described before, we used the KEGG database for pathway matching. We annotated metabolites to KEGG compound IDs, generating KEGG orthology tables from 16S rRNA-based microbiome data, using PICRUSt. Then, we matched KEGG IDs with KEGG pathways. In this step, out of 188 metabolites, in targeted metabolomic data sets, only 74 could be annotated to KEGG compound IDs, and matched to distinct pathways. KEGG orthology also excluded those that were not matched to pathways, leaving only 3394 out of 6241 KEGG orthologs. Despite this limitation, HisCoM-MnM successfully discovered HCC-related pathways.

## 4. Materials and Methods

### 4.1. HCC and LC Data Sets

We performed integrated analysis of microbiome and metabolomic data originating from our previous studies [23,24]. We considered data sets consisting of 103 HCC patients and 97 LC patients who had both microbiome and metabolomic data. Among them, 100 patients were from Seoul National University Hospital (Seoul, Korea), and the others from Ajou University Hospital (Suwon, Korea). This study was approved by the institutional review board of Seoul National University Hospital (IRB No. 1704-021-843), and was conducted in accordance with the Declaration of Helsinki principles. The samples provided by the Korea Biobank Network were obtained, with informed consent, according to IRB-approved protocols.

### 4.2. Microbiome Data

For microbiome data, circulating cell-free DNA was extracted for 16S rRNA gene analysis from serum extracellular vesicles (EVs) [23]. First, we processed a 16S rRNA sequencing data set, via a closed-reference operational taxonomic unit (OTU)-picking pipeline, using QIIME [41], against the Greengenes reference database (gg_13_5). OTUs with all sample zero counts were removed from the OTU table. After filtering, a minimum of 731 and a maximum of 46,024 reads per sample were obtained. The OTU table was rarefied to 3000 reads, with 13 samples having low numbers of read counts (< 3000) being filtered.

For microbiome data, normalization of the OTU table was performed by dividing each OTU by the known or predicted 16S copy number abundance. Next, we generated a functional table, using PICRUSt [42], to predict metagenome contents. The output is a table of KEGG orthology (KO) abundances. The resulting table was normalized using MUSiCC [43]. Finally, 3394 unique KEGG orthologs were mapped to 356 pathways, using the KEGG pathway database.

### 4.3. Metabolomic Data

For metabolomic data, targeted metabolomic analysis using the AbsoluteIDQ^®^ p180 kit (BIOCRATES Life Science AG, Innsbruck, Austria), with liquid chromatography and tandem mass spectrometry, was used to quantify 188 metabolites [24]. We filtered out metabolites with high zero proportion in samples, and normalized by autoscaling for each metabolite.

For metabolomic data, first, we mapped each metabolite to the KEGG COMPOUND ID database, using MetaboAnalyst [44], and checked matching results using databases such as the Human Metabolome Database (HMDB). Finally, 74 unique metabolites were mapped to 126 KEGG pathways.

### 4.4. Merging Metagenomic and Metabolomic Data, Using Pathway Information

For the pathway analysis, we merged metagenomic and metabolomic data sets using KEGG pathway information [45]. KEGG orthologs and metabolites that mapped to the same pathways were grouped together. There were 111 common pathways between 356 metagenomic pathways and 126 metabolomic pathways. Each common pathway consisted of at least one KEGG orthology and metabolite, and the normalized value for each KEGG orthology and metabolite were used as an input data set for HisCoM.

### 4.5. Hierarchical Structural Component Model for Pathway Analysis of Microbiome and Metabolome (HisCoM-MnM)

**Figure 4:**
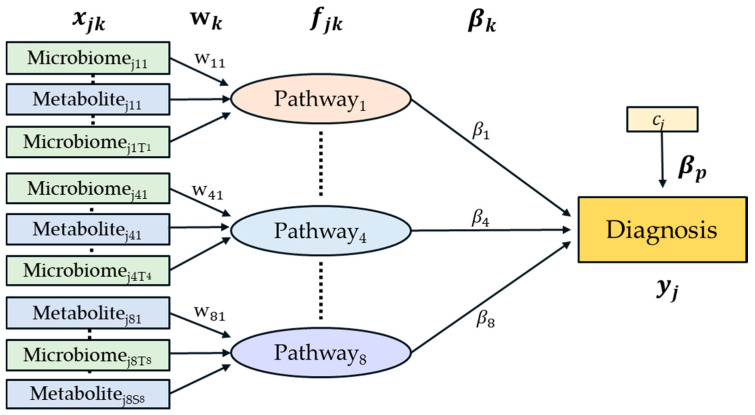
A diagram of the HisCoM-MnM model. Green rectangles represent metabolic genes predicted from microbiome data and blue rectangles represent metabolites. Each pathway consists of metabolic genes and metabolites.

After merging two different types of data sets, pathway analysis could be performed using HisCoM-MnM. The structure of the proposed model is given in Figure 4. This model extends the pathway analysis model of rare variants [17], in that pathways are defined as a weighted component of a set of KEGG orthologs and metabolites.

Let $y_{j}$ denote the phenotype of the jth subject (j=1,…,N). Let K be the number of pathways and $T_{k}$ and $S_{k}$ be the number of KEGG orthologs and metabolites, respectively, in the kth pathway $\left(k=1,\ldots ,K;t=1,\ldots ,T_{k};s=1,\ldots ,S_{k}\right)$. Denote $x_{jkt}$ and $x_{jks}$ as the respective tth and sth KEGG orthology and metabolite in the kth pathway, for the jth subject. Let $w_{kt}$ and $w_{ks}$ denote weights assigned to $x_{jkt}$ and $x_{jks}$. Let $f_{jk}$ be a latent variable representing the main effect of the kth pathway, which is defined as a weighted sum of $T_{k}$ KEGG orthologs and $S_{k}$ metabolites, such that:

(1) $$f_{jk}=\sum_{t=1}^{T_{k}}x_{jkt}w_{kt}+\sum_{s=1}^{S_{k}}x_{jks}w_{ks}$$

Let $\beta_{k}$ denote the coefficient connecting the kth pathway to the phenotype. Let $c_{j}$ denote the covariate of the jth subject, and $\beta_{p}$ denote the coefficients of covariates. Then, the relationship between the binary phenotype and latent variables are established such that:

(2) $$logit\left(\pi \right)=\;\beta_{0}+\overset{K}{\underset{k=1}{\sum }}\left[\overset{T_{k}}{\underset{t=1}{\sum }}x_{jkt}w_{kt}+\overset{S_{k}}{\underset{s=1}{\sum }}x_{jks}w_{ks}\right]\beta_{k}+c_{j}\beta_{p}=\beta_{0}+\overset{K}{\underset{k=1}{\sum }}f_{jk}\beta_{k}+c_{j}\beta_{p}$$

In Figure 4, the first and second layers consist of rectangles and circles that represent the observed variables and latent variables, respectively. As described above, each pathway is defined as a latent variable constructed by a weighted sum of its observed variable consisting of KEGG orthologs and metabolites.

To estimate the model parameters, we used the alternating least squares (ALS) algorithm, which alternates two steps until convergence. In the first step, the pathway coefficient estimates are updated, in the sense of least squares, for fixing the weight coefficient estimates. In the second step, the weight coefficient estimates are updated in the sense of least squares for fixing pathway coefficient estimates. To control the potential correlation between metabolites and KEGG orthologs, and between pathways, we used a ridge-type penalty. Then, we sought to maximize the following penalized log-likelihood function:

(3) $$\phi =\overset{N}{\underset{j=1}{\sum }}\log p\left(y_{j};\beta_{k},\;\delta \right)-\frac{1}{2}\lambda_{m}\overset{K}{\underset{k=1}{\sum }}\left[\overset{T_{k}}{\underset{t=1}{\sum }}w_{kt}^{2}+\overset{S_{k}}{\underset{s=1}{\sum }}w_{ks}^{2}\right]-\frac{1}{2}\lambda_{p}\overset{K}{\underset{k=0}{\sum }}\beta_{k}^{2},$$

where $p\left(y_{j};\beta_{k},\;\delta \right)$ is the probability distribution for $y_{j}$, and $\lambda_{m}$ and $\lambda_{p}$ are ridge parameters for the metabolite, KEGG orthology, and pathway. The values of $\lambda_{m}$ and $\lambda_{p}$ are determined before applying the parameter estimation procedure. These tuning parameters are chosen based on five-fold cross validation. After parameter estimation, to test the pathway and each KEGG orthology and metabolite’s significance, *p*-values were calculated using permutation tests, by generating 100,000 permuted phenotypes.

## 5. Conclusions

In conclusion, we have developed an innovative method, HisCoM-MnM, applied it to 16S rRNA-based microbiome data, and targeted metabolomic data from HCC and LC patients to identify HCC-related pathways, and KEGG orthologs and metabolites. We found that HisCoM-MnM can discover HCC-related pathways that are consistent with previous findings. Further studies with other etiologies of HCC are warranted, to validate the generalizability of our identification of specific pathology-related pathways.

## Figures and Tables

**Table 1:** Basic characteristics of the subjects in each HCC (hepatocellular carcinoma) and LC (liver cirrhosis) group.

Variable	HCC	LC	*p*-Value
	(*n* = 103)	(*n* = 97)	
**Age, Years**	59 (52–64)	57 (49–62)	0.08
**Male**	77 (74.76%)	68 (70.10%)	0.53
**Etiology of Liver Disease**			<0.001
HBV	103 (100.00%)	83 (85.57%)	
HCV	0	12 (12.37%)	
Non-Viral	0	2 (2.06%)	
**Liver Function**			<0.001
Compensated	94 (91.26%)	66 (68.04%)	
Decompensated	9 (8.74%)	31 (31.96%)	
**α-Fetoprotein, ng/ml**	14.8 (4.2–216.8)	3.1 (1.8–9.8)	<0.001
**AJCC TNM Stage**			
I	52 (50.48%)		
II	30 (29.13%)		
III	13 (12.62%)		
IV	8 (7.77%)		

Data are presented as medians with interquartile ranges (IQRs) or numbers (%), unless otherwise indicated. AJCC, the American Joint Committee on Cancer; HBV, hepatitis B virus; HCV, hepatitis C virus; HCC, hepatocellular carcinoma; LC, liver cirrhosis.

**Table 2:** Categories and subcategory of KEGG pathways.

Pathway Category	Subcategory	Number of Pathways	
Metabolism	Biosynthesis of other secondary metabolites	7	24
	Metabolism of other amino acids	7	
	Amino acid metabolism	4	
	Carbohydrate metabolism	2	
	Energy metabolism	1	
	Global and overview maps	1	
	Lipid metabolism	1	
	Metabolism of cofactors and vitamins	1	
Organismal Systems	Digestive system	2	5
	Nervous system	2	
	Excretory system	1	
Human Diseases	Cancer: overview	1	4
	Drug resistance: antimicrobial	1	
	Neurodegenerative disease	1	
	Substance dependence	1	
Environmental Information Processing	Signal transduction	3	3
Cellular Processes	Cell growth and death	1	2
	Cell motility	1	
Genetic Information Processing	Translation	1	1

**Table 3:** Significant pathways, within specific categories and subcategories, related to HCC.

Category	Pathway Category	Sub Category	Name	*p*-Value
**Cancer**	Human Diseases	Cancer: overview	Central carbon metabolism in cancer	2.0 × 10^−5^
	Human Diseases	Cancer: overview	Choline metabolism in cancer	2.54 × 10^−3^
**Amino Acid Metabolism**	Metabolism	Amino acid metabolism	Glycine, serine and threonine metabolism	2.0 × 10^−4^
	Metabolism	Amino acid metabolism	Valine, leucine and isoleucine biosynthesis	8.0 × 10^−5^
	Metabolism	Amino acid metabolism	Alanine, aspartate and glutamate metabolism	9.80 × 10^−4^
	Metabolism	Amino acid metabolism	Valine, leucine and isoleucine degradation	3.18 × 10^−3^
	Metabolism	Metabolism of other amino acids	D-Glutamine and D-glutamate metabolism	2.0 × 10^−5^
	Metabolism	Metabolism of other amino acids	D-Alanine metabolism	2.0 × 10^−5^
	Metabolism	Metabolism of other amino acids	Glutathione metabolism	1.40 × 10^−4^
**Lipid Metabolism**	Metabolism	Global and overview maps	Fatty acid metabolism	6.0 × 10^−5^
	Metabolism	Lipid metabolism	Fatty acid degradation	1.40 × 10^−4^
	Metabolism	Lipid metabolism	Glycerophospholipid metabolism	1.18 × 10^−3^
	Metabolism	Lipid metabolism	Primary bile acid biosynthesis	3.78 × 10^−3^
	Metabolism	Lipid metabolism	Linoleic acid metabolism	0.029

## Glossary

Microbiota	microorganisms present in a defined environment [46]
Metagenome	the collection of genomes and genes sequenced from members of a microbiota [46]
Microbiome	the entire habitat, including the microorganisms, their genomes, and the surrounding environmental conditions [46]
Metabolome	collection of all metabolites present in the cell that are participants in general metabolic reactions [47]
Omics	large-scale study of data representing an entire set of molecules, such as proteins, lipids, or metabolites, in a cell, organ, or organism
Multiomics	an approach that examines multiple omics data sets such as genomics, proteomics, transcriptomics, metabolomics, and microbiomics
